# Missed nursing care and its relationship with nurses’ moral sensitivity: a descriptive-analytical study

**DOI:** 10.1186/s12912-024-01854-8

**Published:** 2024-03-13

**Authors:** Naiier Ahansaz, Mohsen Adib-Hajbaghery, Rahim Baghaei

**Affiliations:** 1 Departmentof Medical Surgical Nursing, Trauma Nursing Research Center, Faculty of Nursing and Midwifery, Kashan University of Medical Sciences, Kashan, Iran; 2 Trauma Nursing Research Center, Faculty of Nursing and Midwifery, Kashan University of Medical Sciences, 5th of Qotb –e Ravandi Blvd, P.O.Box: 8715981151, Kashan, Iran; 3 Patient Safety Research Center, Clinical Research Institute, Urmia University of Medical Sciences, Urmia, Iran

**Keywords:** Missed nursing care, Nursing, Moral sensitivity

## Abstract

**Background:**

Missed care rates are an indicator of healthcare quality. Missed nursing care can reduce patient safety and lead to adverse events. Moral sensitivity enables nurses to interpret and respond to clients’ needs according to ethical principles. Despite the importance of moral sensitivity and its role in the quality of care, the relationship between nurses' moral sensitivity and missed nursing care has not been extensively studied. This raises the question of whether there is an association between nurses' moral sensitivity and missed care. This study was conducted to investigate the relationship between nurses’ moral sensitivity and missed nursing care in the medical and surgical departments of Imam Khomeini Hospital in Urmia, Iran.

**Methods:**

A cross-sectional study was conducted in 2022, on 202 nurses working in the medical and surgical departments of Imam Khomeini Medical Education Center, Urmia, Iran. Stratified random sampling was used to select the participants. A questionnaire on demographic characteristics, Kalisch's missed care questionnaire, and Lutzen's moral sensitivity questionnaire were used to collect data. Data were analyzed using the Pearson correlation test and linear regression analysis.

**Results:**

Most participants (52.97%) showed moderate moral sensitivity. Nurses’ mean moral sensitivity and total missed care scores were 149.07 ± 21.60 and 59.45 ± 4.87, respectively. Pearson correlation test showed no significant correlation between moral sensitivity scores and the total missed care scores (*r* = 0.041, *p* = 0.152). However, in the regression analysis, moral sensitivity (β = 0.213, *p* < 0.001), age (β = 0.131, *p* < 0.001), working in rotating shifts (β = 0.183, *p* < 0.001), monthly income (β = 0.079, *p* = 0.004), work experience (β = 0.030, *p* = 0.010), and monthly work hours (β = 0.247, *p* = 0.010) influenced missed care. Approximately, 0.98% of the variance in the missed care was explained by these variables.

**Conclusion:**

Our nurses reported moderate levels of moral sensitivity and a concerning level of missed care. Missed care can have detrimental effects on patient safety. Therefore, nursing managers must address this issue promptly.

## Background

It has been shown that approximately 13.5% of patients experience an unfortunate event during their hospitalization. Furthermore, that approximately three-quarters of nurses missed at least one care during their last work shift [1]. In some hospitals, this rate reaches 100% of nurses [2]. The estimated cost of unsafe care accounts for about 10% of total healthcare expenditure [3].

Missed nursing care refers to the omission or delay in providing essential nursing interventions or activities that are necessary and expected to ensure patient safety, wellbeing, and recovery [4]. This may include failure to administer medications on time, neglecting necessary hygiene or comfort measures, or failing to respond promptly to patient calls [5, 6]. Missed care can occur in primary care (i.e. the fundamental care needed to maintain a patient’s health, safety, and well-being such as care related to medication administration, ensuring good hygiene, assisting with feeding, monitoring vital signs, and addressing any immediate health concerns), in supportive care (i.e. attention to the emotional, psychological, and social needs of patients), and in secondary care (i.e. emotional support for the patient's family, mouth care, discharge planning and patient education, focused reevaluation of the patient, evaluation of drug effects, and attending interdisciplinary caring conferences) [7].

Missed nursing care can reduce patient safety and lead to adverse events such as falls, phlebitis, nosocomial infections, medication errors, pressure ulcers, urinary tract infections, delirium, pneumonia, increased pain and discomfort, prolonged hospitalization, decreased satisfaction, delayed hospital discharge, readmissions within 30 days, physical disability, and patient death [5, 8–10]. Missed care also affects human resources and healthcare organizations. These effects include increased workload and psychological pressure on nurses, increased conflict and stress in the work environment, decreased job satisfaction among nurses, and an increased propensity to leave the profession. Missed care can also cause people to distrust healthcare providers and the healthcare system [3, 5, 8, 11, 12].

Missed care rates are an indicator of quality [13]. However, nurses’ caring behavior and the quality of care they provide cannot be separated from ethics. Ignoring ethical issues can cause obvious and hidden underperformance [14, 15]. According to the four-component model, nurses' behavior and actions are affected by their moral sensitivity, moral judgment, moral motivation, and moral behavior [16]. Nurses' moral sensitivity plays a crucial role in their decision-making and actions regarding patient care [12]. It refers to their ability to recognize ethical dilemmas, perceive the moral aspects of a situation, and respond ethically by considering the patient's best interest [2]. Nurses' moral sensitivity is influenced by various factors, including individual values, beliefs, professional standards, workload, staffing ratios, organizational culture, available resources, and the broader healthcare context [2, 17–19]. Moral sensitivity is necessary for ethical practice and enables nurses to interpret and respond to clients’ needs according to ethical principles. It, therefore, improves the quality of care, strengthens nurses’ professional performance, increases their responsibility, and encourages them to perform their nursing roles correctly. Additionally, patients' trust and satisfaction with nurses improve, and nurse-patient communication is enhanced [18, 20–22]. Moral sensitivity develops people's moral reasoning, ensuring that they have the correct moral perspective and behavior when faced with moral challenges [23].

When examining nurses' moral sensitivity, studies have reported varying results. Two studies from Iran reported that most nurses had moderate moral sensitivity [24, 25]. Filipova (2009) found that nurses working in one of the Midwestern state of the US had low moral sensitivity [26]. Some studies suggest that there is a correlation between nurses’ moral sensitivity and caring behavior [21, 27]. A study by Shahveli et al. (2017) showed a positive correlation between nurses' moral sensitivity and patients' satisfaction with the quality of nursing care [28]. Furthermore, Golnar et al. (2020) reported that higher moral sensitivity was associated with higher awareness of medical errors and error reporting [29]. However, Izadi et al. (2013) and Amiri et al. (2016) found no association between nurses’ moral sensitivity and their caring behaviors or quality of care [30, 31].

Despite the importance of moral sensitivity and its role in the quality of care, the relationship between nurses' moral sensitivity and missed nursing care has not been extensively studied. Due to the scarcity of research in this area, the question arises whether there is a link between nurses’ moral sensitivity and missed nursing care. Understanding the relationship between nurses' moral sensitivity and missed nursing care is essential for developing strategies and interventions to improve patient care outcomes. Therefore, this study was conducted to investigate the relationship between nurses’ moral sensitivity and missed nursing care in the medical and surgical departments of Imam Khomeini Hospital in Urmia, Iran. It is hoped that this study can help plan the education and training of capable nurses in patient care, reduce the number of missed care, and improve the quality of care.

## Methods

### Study design and setting

This cross-sectional study was conducted at Imam Khomeini Medical Education Center in Urmia, Iran, in 2022. Data collection was conducted from November 22, 2022 to December 23, 2022.

### Participants and sample size

Participants were nurses working in the medical and surgical wards of the Imam Khomeini Medical Education Center in Urmia, Iran. Due to the lack of similar studies at the time of study design, Morgan's table was used to determine the sample size. The total number of nurses working in the medical and surgical wards of the above-mentioned hospital was 378, so according to Morgan's table, 191 nurses were required to be included in the study. However, we increased the sample size to 210 to compensate for possible dropouts. A list of eligible nurses working in the medical and surgical wards of the hospital was then prepared from the hospital's nursing office, and stratified random sampling was conducted to ensure the representativeness of the sample. To this end, the researcher first estimated the number of samples needed from each ward according to the ratio of the number of nurses in the ward to the total sample size required. The required number of samples was then recruited from each ward using a random number table. The first researcher referred to the preselected eligible nurses, introduced herself, explained the objectives of the study, and invited them to participate in the study. If a nurse declined to participate, another nurse from the same ward was randomly selected. Of the 235 eligible nurses, 22 refused to participate and 3 were transferred to other medical centers. A total of 210 people returned completed questionnaires. Of these, 10 were excluded because they had filled out the questionnaires incompletely, and finally 202 questionnaires were finally analyzed.

Inclusion criteria included direct contact with the patients (first line of communication and treatment), a bachelor's degree or higher, willingness to participate in the study, and at least six months of clinical work experience. Nurses were excluded if they withdrew from the study while completing the questionnaires or if answered to the questionnaires incompletely.

### Measurements

Three questionnaires were used to collect data, including a questionnaire on demographic and professional characteristics, Kalisch's missed care questionnaire, and Lutzen's moral sensitivity questionnaire.

The demographic and professional characteristics questionnaire included items on nurses’ gender, age, marital status and children, employment status, type of work shift, education level, amount received, work experience, monthly working hours, previous attendance at ethics workshops, time elapsed since attending the last ethics workshop, average number of patients per work shift, and willingness to leave the job.

Kalisch's missed care questionnaire (MISSCARE) was developed by Kalisch in 2006, and then psychometrically validated and revised in 2009. Internal consistency of the questionnaire was confirmed by a Cronbach's alpha of 0.87 [32]. The questionnaire has two parts. Part A assesses missed nursing care activities and part B assesses reasons behind missed care. In the present study, we used the part A. This part consists of 24 items scored on a 5-point Likert scale, including "0: Never forgotten,” “1: Seldom forgotten,” “2: Sometimes forgotten,” “3: Often forgotten,” and “4: Always forgotten." The total score ranges between 0 and 96. Higher scores indicate more missed care. Hosseini et al. (2022) have translated the MISSCARE into Persian and psychometrically validated it. Through factor analysis, they categorized the missed care activities (part A) into three categories: primary, secondary, and supportive care. They reported that the Cronbach’s alpha for part A was 0.933 [7].

Lutzen's Moral Sensitivity Questionnaire (MSQ) consists of 30 items designed to examine nurses’ moral sensitivity when providing clinical services. The items are distributed across six dimensions, namely respect for client autonomy (7 items), interpersonal communication (4 items), respect for rules (4 items), experience of ethical conflicts and challenges (3 items), benevolence (4 items), and applying ethical concepts (5 items). Furthermore, there are 3 items as others. The items are scored on a 7-point Likert scale, from “1: Absolutely agree” to 7: Absolutely disagree.” The total score of the questionnaire ranges from 30 to 210. Higher scores indicate lower levels of moral sensitivity. Scores 30–90, 91–150, and 151–210 are considered high, moderate, and low moral sensitivity, respectively. Hassanpoor et al. (2011) translated and validated the MSQ in Iran. They validated the MSQ through the content validity method, then administered it to 20 nurses and reported that its Cronbach alpha coefficient was 0.81 [33].

The researcher instructed the participants on how to answer to the questionnaires and asked them to complete them individually, in a private setting, and to return the completed questionnaires to the researcher at her next visit on the same day or the next day. In the latter case, participants were given the researcher's telephone number for coordination purposes.

### Ethical considerations

The researchers received approval from the ethics committee of Kashan University of Medical Sciences (Code:IR.KAUMS.NUHEPM.REC.1401.058) and Imam Khomeini Medical Training Center in Urmia, Iran, ensuring that the study adheres to ethical standards and guidelines. Nurses were provided with detailed information about the purpose of the study, the nature of their involvement, and the potential impact. They were allowed to ask questions and voluntarily agreed to participate. Written informed consent was taken from all the participants. To protect nurses' privacy, all data collected were anonymized. Personal information was kept confidential, and the identities of participants were not disclosed in any reports or publications. All variables in the questionnaire were handled using specific codes. While answering the questionnaires, the researcher was available for the participants to answer their questions (if any).

### Data analysis

The data were analyzed using descriptive and inferential statistics in SPSS software version 16. Before entering the data into the statistical software, we carefully reviewed the collected data for any missing values or inconsistencies. No missing data was found in this study. Any data entry errors were also identified and corrected through a thorough data cleaning process. Descriptive statistics such as frequency, percentage, mean, and standard deviation are described. The Pearson's correlation test was used to examine the correlation between moral sensitivity scores (as exposure) and missed care (as outcome). Linear regression analysis was also used to examine the factors affecting missed care as an outcome variable. Moral sensitivity and demographic characteristics were entered into the regression model as independent variables. To enter the variables into the regression, firstly forward method was used. Then, all variables with a significance level of ≤ 0.2 were reentered into the model using the backward method. None of the quantitative variables were categorized or transformed for entering the regression analysis. However, we transformed the categorical variables into dummy variables.

## Results

A total of 235 nurses met the eligibility criteria. Of them, 22 declined to participate and 3 were transferred to other medical centers. Finally, 210 nurses returned completed questionnaires but 10 of them were incomplete. Therefore, 202 questionnaires were analyzed (response rate 96.19%). The majority of the nurses (56.4%) were male, 51.5% were married, and 96% had a bachelor's degree. Most of the participants (97%) worked in rotating shifts, and 68.3% had less than 5 years of work experience. The participants had a mean age of 30.95 ± 6.03 years and a mean work experience of 4.26 ± 3.88 years. They worked an average of 166.39 ± 24.60 h per month and managed 11.04 ± 1.13 patients in their previous shift (Table 1).

**Table 1:** Demographic characteristics of the nurses participating in the study

Variables		Number (%)
Gender	Male	114 (56.4)
	Female	88 (43.6)
Having a child	Yes	84 (41.6)
	No	118 (58.4)
Marital status	Single	104 (51.4)
	Married	84 (41.6)
	Divorced	10 (5)
	Widow	4 (2)
Income	Low	15 (7.4)
	Medium	100 (45.9)
	Up	76 (37.6)
	Rich	11 (5.4)
Education level	Bachelor	194 (96)
	Master	8 (4)
Shift work	Fix	6 (3)
	Rotating	196 (97)
Willingness to leave the job	Yes	101 (50)
	No	101 (50)
Work experience	5 >	138 (68.3)
	5–10	52 (25.7)
	10 <	12 (5.9)
	Mean ± SD	4.26 ± 3.88
Age, Mean ± SD		30.95 ± 6.03
Monthly working hours, Mean ± SD		166.39 ± 24.60
Number of patients in the last work shift, Mean ± SD		11.04 ± 1.13

The nurses’ mean moral sensitivity was 149.07 ± 21.60, and their mean total missed care score was 59.45 ± 4.87 (Table 2). Most participants (52.97%) showed moderate moral sensitivity, while 23.76% and 23.26% had low and high moral sensitivity, respectively.

**Table 2:** Moral sensitivity and missed care scores of the participants (*n* = 202)

**Variables**	**The number of items per subscale**	**Range of scores for each subscale (Min, Max)**	**Total possible score of the questionnaires**	**Mean**	**Standard deviation**
Moral sensitivity	30	30, 203	30–210	149.07	21.60
Respect for client autonomy	7	13, 48	7–49	36.68	5.87
Interpersonal communication	4	9, 27	4–28	19.33	3.24
Respect for rules	4	9, 28	4–28	19.47	3.24
Experience of ethical conflicts	3	6, 20	3–21	14.48	2.59
Benevolence	4	9, 28	4–28	19.63	3.34
Applying ethical concepts	5	12, 34	5–35	25.45	4.31
Others	3	6, 20	3–21	13.94	2.41
Missed care					
Primary care	13	20, 37	0–52	26.10	2.58
Supportive care	5	8, 18	0–20	13.42	1.81
Secondary care	6	11, 22	0–24	19.25	2.39
Total score of missed care	24	49, 78	0–96	59.45	4.87

The Pearson correlation showed no association between moral sensitivity and the total score of missed care (*r* = 0.041, *p* = 0.152) (Table 3).

**Table 3:** The correlation between moral sensitivity and missed care

Variables		Pearson correlation	*P*-value
Moral sensitivity	Primary care	0.090	0.196
	Supportive care	-0.125	0.052
	Secondary care	0.067	0.331
	Total score of missed care	0.041	0.152

In regression analysis, moral sensitivity, age, working in rotating shifts, monthly income, work experience, and monthly work hours influenced missed care. The two factors with the greatest effect were monthly working hours and moral sensitivity, respectively (Table 4).

**Table 4:** Results of the regression analysis to determine the factors influencing missed care

Model	Unstandardized Coefficients		Standardized Coefficients	T	Sig	Adjusted R Square
	B	Std. Error	Beta			
Moral sensitivity	.084	.020	.213	4.295	< 0.001	0.988
Monthly working hours	.087	.016	.247	5.352	< 0.001	
Rotating shift	11.018	2.439	.183	4.518	< 0.001	
Age	.247	.073	.131	3.409	< 0.001	
Bachelor’s degree	8.019	2.194	.132	3.655	< 0.001	
Income	1.880	.639	.079	2.942	0.004	
Work experience	.306	.118	.030	2.586	0.010	

Dependent Variable: Missed care

## Discussion

More than half of the nurses participating in this study demonstrated moderate levels of moral sensitivity. Consistent with our findings, Sharifi et al. (2020) also assessed the moral sensitivity of nurses working in public hospitals of Kashan, Iran and reported that their nurses scored at a moderate level [34]. Similar results were reported by Dalvand et al. (2019) who studied nurses in Khorramabad, Iran [24], HosseinAbadiFarahani et al. (2019), who studied Iranian critical care nurses [35], and Jasemi et al. (2020), who studied the moral sensitivity of final year nursing students studying at the Urmia nursing school [36]. Ertuğ et al. (2014) also studied the moral sensitivity of 111 nurses working in two public and university hospitals in Ankara, Turkey. Nurses in the latter study scored approximately half of the moral sensitivity score [37], indicating a moderate level of moral sensitivity. The moderate level of moral sensitivity in the current study suggests that while nurses possess a certain degree of moral sensitivity, there is room for further development and improvement. As frontline caregivers, nurses are in constant close contact with patients and their families, dealing with sensitive and important issues. They should observe patient rights and privacy, advocate for patient rights, protect patients, comply with professional ethics, and meet patients' needs, even when they are dying. Therefore, nurses need to be very sensitive and ethically attentive and must exhibit a high level of moral sensitivity. They need to have strong moral strength and choose the right course of action in all situations based on ethical issues. This will have a positive impact on the quality of care they provide [38]. Therefore, efforts should be made to enhance nurses’ moral sensitivity through targeted training programs and ongoing education.

Based on our study, the nurses scored an average of 59.45 out of 96 for missed care, indicating a concerning level of missed care in the current healthcare setting. In a recent study of 215 nurses working in the medical-surgical wards of eight public and private hospitals in Tabriz, Iran, 72% of nurses reported missing at least one nursing care on their last shift [10]. Another study in hospitals of Birjand, Iran, also used the same instrument as ours to assess missed care and reported that nurses scored 82.04, signifying a high level of missed care [39]. Missed care refers to essential healthcare activities that are not completed or delayed, potentially compromising patient outcomes [4]. Although the mean missed care score in the current study was lower than the study conducted in Birjand [39], a score over the midpoint of the scale, suggests that there is room for improvement in ensuring that all necessary care tasks are consistently carried out. It is important to address this issue promptly as missed care can have detrimental effects on patient safety, quality of care, and overall patient satisfaction. Further research is needed to identify the specific reasons behind the missed care in the current healthcare setting and to develop strategies that promote timely and comprehensive delivery of care by nurses.

Correlation analysis revealed no significant association between the scores of moral sensitivity and missed care. Some studies have examined the correlation between moral sensitivity and variables such as nurses’ caring behavior, quality of care, respect for patient rights, or nurses’ ethical behaviors and have reported inconsistent findings. For instance, in a study conducted in Bandar Abbas, Iran, most nurses exhibited moderate levels of moral sensitivity and caring behavior. Furthermore, no significant correlation was found between moral sensitivity scores and caring behavior scores. However, nurses who showed more respect for patient autonomy had attended more medical ethics seminars or workshops [30]. Amiri et al. (2016) also found no significant relationship between nurses' ethical sensitivity scores and quality-of-care scores [31]. On the other hand, Mahdiyoun et al. (2016), studied the correlation between nurses' moral sensitivity and respect for patients' rights. They reported a moderate level of both nurses' moral sensitivity and respect for patient rights, and a positive correlation between these two variables [40]. Ghasemi et al. (2017) also examined the correlation between moral sensitivity, mental health, and ethical behavior among nurses in Ardabil, Iran. They found a positive relationship between moral sensitivity and ethical behavior [23]. Although the above-mentioned studies did not directly assess the correlation between moral sensitivity and missed care, it can be assumed that caring behaviors and quality of care are connected with missed care. The inconsistencies between the studies can be attributed to methodological differences and contextual factors. Therefore, further studies should directly address the correlation between moral sensitivity and missed care.

In the present study, linear regression results showed that in addition to moral sensitivity, missed care was affected by various factors, including the nurse's age, income, level of experience and education, working hours per month, and working in rotating shifts. Among these variables, the largest effects were related to monthly work hours and moral sensitivity. Cho et al. (2012) in a cross-sectional study investigated the effect of nurses' work schedules on missed care. They reported that the number of hours worked per day and lack of rest were significantly linked with missed care [41]. Kalisch's model suggests that nurse-related factors, such as the number and type of nurses on the shift, their competence, work experience, and education, are related to the amount of missed care [42]. However, a study reviewing factors related to missed care has found that organizational factors and characteristics, ward characteristics, and the level of teamwork between nurses and other health service providers such as physicians, have the greatest impact on impact on missed care [43]. By reviewing studies, Amrolahi-mishavan concluded that the amount of missed care is influenced by nurses' characteristics, workload, staffing levels, job satisfaction, available resources for care, communication, teamwork, work environment conditions, management, and the use of technology in providing care [44]. The results of our study, along with these findings, suggest that individual characteristics, workload, and environmental and organizational factors significantly affect missed care. Therefore, to reduce missed care, hospital managers should aim to reduce nurses' workload, improve their working conditions, provide training, and promote nurses' moral sensitivity.

In this study, we made efforts to minimize potential sources of bias and ensure the validity of our findings. To address selection bias, we employed a stratified random sampling method, which helped reduce the likelihood of systematic bias in participant selection. To minimize measurement bias, we used well-established and validated instruments for data collection. To minimize the possibility of information bias, the researcher who collected the data underwent rigorous training to ensure consistency and accuracy in administering the questionnaires. Clear instructions were also provided to the participants to encourage truthful and impartial responses. However, despite our efforts, there are a few limitations to consider. First, the study was conducted at a single medical center in Urmia, Iran, which may limit the generalizability of the findings to other settings. Additionally, the study design was cross-sectional, which restricts our ability to establish causality between moral sensitivity and missed care. Additionally, despite our best efforts to ensure confidentiality, as with any survey-based research, there is the potential for response bias and social desirability bias. Furthermore, although we considered the potential confounding effects of demographics in the data analysis, the effects of unmeasured or unknown confounders cannot be entirely ruled out. For example, factors such as a nurse's social class, cultural background, and religious beliefs, as well as the work environment culture, may influence ethical sensitivity and missed care. However, these influences were beyond the researcher's control.

## Conclusion

Our nurses reported moderate levels of moral sensitivity and a concerning level of missed care. We also found a connection between missed care and moral sensitivity, as well as working hours, working in rotating shifts and income. These findings highlight the need to promote nurses' moral sensitivity and provide the necessary conditions to reduce missed care. Missed care can have detrimental effects on patient safety, quality of care, and overall patient satisfaction. Therefore, nursing managers must address this issue promptly. To decrease missed care, nurses' moral sensitivity can be strengthened through in-service education, role modeling, provision of ethical guidelines, and encouraging reflective practice. Additionally, decreasing nurses' workload and monthly working hours, and improving their income can also be helpful. Nursing authorities should also periodically assess nurses' moral sensitivity levels and influencing factors, as well as the prevalence of missed care in clinical settings. This information can help them design and implement appropriate interventions, such as training and supervision, to increase nurses' moral sensitivity and reduce missed care.

## Data Availability

The datasets used and/or analyzed during the current study are available from the corresponding author on reasonable request.
